# Diallyl thiosulfinate enhanced the anti-cancer activity of dexamethasone in the side population cells of multiple myeloma by promoting miR-127-3p and deactivating the PI3K/AKT signaling pathway

**DOI:** 10.1186/s12885-021-07833-5

**Published:** 2021-02-06

**Authors:** Wenfeng He, Yonghui Fu, Yongliang Zheng, Xiaoping Wang, Bin Liu, Junquan Zeng

**Affiliations:** 1 Jiangxi Key Laboratory of Molecular Medicine, The Second Affiliated Hospital of Nanchang University, Nanchang, 330006 Jiangxi Province China; 2 Department of Psychiatry, Jiangxi Mental Hospital, Nanchang, 330029 Jiangxi Province China; 3 Department of Hematology, The Affiliated Hospital of Jinggangshan University, ji’an, 343000 Jiangxi Province China; 4 Comprehensive teaching and research office, Ji’an College, ji’an, 343000 Jiangxi Province China; 5 Department of internal medicine, Jinggangshan University, ji’an, 343009 Jiangxi Province China

**Keywords:** Multiple myeloma, Side population cells, Diallyl trisulfide, Dexamethasone, miR-127-3p, PI3K signaling pathway

## Abstract

**Background:**

Side population (SP) cells, which have similar features to those of cancer stem cells, show resistance to dexamethasone (Dex) treatment. Thus, new drugs that can be used in combination with Dex to reduce the population of SP cells in multiple myeloma (MM) are required. Diallyl thiosulfinate (DATS, allicin), a natural organosulfur compound derived from garlic, has been shown to inhibit the proliferation of SP cells in MM cell lines. Therefore, we investigated the effect of a combination of DATS and Dex (DAT + Dex) on MM SP cells.

**Methods:**

SP cells were sorted from MM RPMI-8226 and NCI-H929 cell lines using Hoechst 33342-labeled fluorescence-activated cell sorting. The growth of SP cells was evaluated using the cell counting kit-8 assay. Cell cycle and apoptosis assays were conducted using a BD Calibur flow cytometer. miRNA expression was measured using quantitative reverse transcription-polymerase chain reaction. Phosphoinositide 3-kinase (PI3K), phosphorylated AKT (p-AKT), AKT, p-mechanistic target of rapamycin (mTOR), and mTOR levels were measured using western blot analysis.

**Results:**

Our results showed that the combination of DATS+Dex inhibited sphere formation**,** colony formation, and proliferation of MM SP cells by inducing apoptosis and cell cycle arrest in the G1/S phase. In addition, the combination of DATS+Dex promoted miR-127-3p expression and inhibited PI3K, p-AKT, and p-mTOR expression in SP cells. Knockdown of miR-127-3p expression weakened the effect of DATS+Dex on cell proliferation, colony formation, apoptosis, and cell cycle of MM SP cells. Additionally, knockdown of miR-127-3p activated the PI3K/AKT/mTOR signaling pathway in MM SP cells cotreated with DATS+Dex.

**Conclusion:**

We demonstrated that cotreatment with DATS+Dex reduced cell proliferation, promoted apoptosis, and caused cell cycle arrest of MM SP cells by promoting miR-127-3p expression and deactivating the PI3K/AKT/mTOR signaling pathway.

**Supplementary Information:**

The online version contains supplementary material available at 10.1186/s12885-021-07833-5.

## Background

Multiple myeloma (MM) is a type of cancer that causes abnormal proliferation of plasma cells in the bone marrow [1]. Despite considerable progress in the treatment of MM, the 5-year survival rate of patients with this cancer is only approximately 47%, mainly because of the prevalence of drug resistance and tumor relapse [2], which are associated with the existence of cancer stem cells (CSCs). Side population (SP) cells have many similar features to those of CSCs and can be separated from main population (MP) cells using flow cytometry-based sorting with Hoechst 33342 staining [3]. SP cells have important pathophysiological and clinical characteristics that strongly correlate with tumor relapse and drug resistance [4]. Dexamethasone (Dex) is a drug commonly used for the treatment of MM [5], but SP cells show resistance to Dex treatment [6].

Thus, there is a need to discover new safer drugs that can be used in combination with Dex to reduce SP cell population in MM. Diallyl thiosulfinate (DATS, allicin) is a natural organosulfur compound derived from garlic that has been investigated as potential anticancer drug [7, 8]. DATS inhibits proliferation and promotes apoptosis of SP cells in MM [9]. Additionally, several studies have shown that DATS-induced apoptosis involves the phosphoinositide 3-kinase (PI3K)/AKT/mechanistic target of rapamycin (mTOR) signaling pathway [10–12]. However, whether DATS can enhance the inhibitory effect of Dex on the proliferation of SP cells remains unclear.

MicroRNAs (miRNAs), small noncoding RNA molecules that modulate gene expression, have been confirmed to have prognostic significance in MM [13]. miRNAs also regulate MM growth, metastasis, and chemotherapy sensitivity by inhibiting target gene expression [14–16]. A previous study found that miR-451 promoted the proliferation and colony formation of SP cells in MM [17]. However, whether miRNAs could be beneficial as a cotreatment with DATS and Dex (DATS+Dex) in regulating SP cell proliferation remains unclear.

In this study, we investigated the effects of cotreatment with DATS+Dex on the proliferation, cell cycle, and apoptosis of MM SP cells. Subsequently, we analyzed the potential role of miRNA and the PI3K/AKT/mTOR signaling pathway in the regulatory mechanism involved in the effect of this combination on MM SP cells.

## Methods

### Cell culture, SP cell separation, and treatments

The human MM RPMI-8226 (CCL-155) and NCI-H929 (CRL-9068) cell lines were obtained from American Type Culture Collection (Manassas, VA, USA) and cultured as previously described [18]. SP cells were sorted from the MM RPMI-8226 and NCI-H929 cell lines using Hoechst 33342-labeled fluorescence-activated cell sorting as previously described [18]. SP cells were separately treated with 10 μg/mL DATS [9], 50 μM Dex (Sigma Aldrich, St Louis, MO, USA) [6], and cotreatment with 10 μg/mL DATS+ 50 μM Dex, and untreated cells were used as a control group.

### Proliferation, colony formation, and sphere formation assays

The growth of RPMI-8226 and NCI-H929 cells was evaluated using the MTS (Beyotime, Shanghai, China) in accordance with the manufacturer’s instructions. A colony formation assay was performed as previously described [19]. For sphere formation assays, SP cells were seeded in ultra-low attachment plates under stem cell conditions by culturing in Dulbecco’s modified Eagle’s medium-F12 culture medium containing 20 ng/mL epidermal growth factor, 10 ng/mL basic fibroblast growth factor, and 1× B-27 supplement, under saturated humidity conditions at 37 °C with 5% CO_2_. The spheres were visualized using microscopy (Olympus, Tokyo, Japan).

### Cell cycle and apoptosis analysis

Cell cycle and apoptosis assays were conducted using the Cell Cycle Detection and apoptosis detection kits (Keygen, Nanjing, China), respectively. The samples were analyzed using a BD Calibur flow cytometer (BD, Franklin Lakes, NJ, USA).

### Western blot analysis

Total protein isolation, concentration determination, and western blotting were performed as previously described [18]. Protein was extracted from SP cells and determined using a protein assay kit (Keygentec, Nanjing, China). Denatured protein samples (20 μg) were separated using 10% sodium dodecyl sulfate-polyacrylamide gel electrophoresis and transferred onto polyvinylidene fluoride membranes (Millipore, Billerica, MA, USA). After blocking, the membranes were incubated at 4 °C overnight with primary antibodies. Then, the membranes were washed and incubated with horseradish peroxidase (HRP)-conjugated goat anti-IgG H&L secondary antibodies (1:20,000) for 2 h at 25 °C. The protein bands were visualized using an enhanced chemiluminescence (ECL) kit (Thermo Scientific, Rockford, IL, USA) and quantified using the Image Lab 6.0 software. The expression of all proteins was normalized to that of glyceraldehyde 3-phosphate dehydrogenase. The blots were incubated with the following primary antibodies: anti-PI3K (1:500), anti-p-AKT (1:500), anti-total AKT (t-AKT, 1:200), anti-mTOR antibody (1:1000), and anti-p-mTOR antibody (1:500) (all from Santa Cruz).

### GEO data analysis and quantitative reverse transcription-polymerase chain reaction (qRT-PCR)

miRNA expression datasets (GSE56163) were downloaded from Gene Expression Omnibus (http://www.ncbi.nlm.nih.gov/geo), a high-throughput gene expression database, and analyzed using GEO2R analysis. miRNA datasets of SP and MP cells in MM were compiled using the GPL11434 miRCURY LNA microRNA Array, 6th generation, and were analyzed using qRT-PCR. Briefly, SP cells were collected, followed by extraction of total RNA using TRIzol reagent (Invitrogen) and reverse transcription to complementary DNA using an ImProm-II reverse transcription system (Promega). The expression of miRNAs and mRNA was determined using SYBR GREEN qPCR Super Mix (Invitrogen) with the U6 gene as the internal reference. All experiments were performed in duplicate and repeated three times. The results are represented as fold-induction, which was calculated using the 2^-ΔΔCT^ method. The primers used to determine the expression of miRNAs are shown in Table 1.

**Table 1:** Primer sequence

Gene	Primer sequence (5′–3′)	Size (bp)
miR-138 F	ACACTCCAGCTGGGAGCTGGTGTTGTGAATC	73
miR-138 R	CTCAACTGGTGTCGTGGA	
miR-3200-5p F	ACACTCCAGCTGGGAATCTGAGAAGGCGCA	72
miR-3200-5p R	CTCAACTGGTGTCGTGGA	
miR-127-3p F	ACACTCCAGCTGGGTCGGATCCGTCTGAGC	72
miR-127-3p R	CTCAACTGGTGTCGTGGA	
U6 F	CTCGCTTCGGCAGCACA	94
U6 R	AACGCTTCACGAATTTGCGT	
ALDH1 F	TCACAGGATCAACAGAGGTTGG	170
ALDH1 R	GCCCTGGTGGTAGAATACCC	
Sox2 F	TACAGCATGATGCAGGACCA	231
Sox2 R	CTCGGACTTGACCACCGAAC	
GAPDH F	GCTCATTTGCAGGGGGGAG	138
GAPDH R	GTTGGTGGTGCAGGAGGCA	

*ALDH1* Aldehyde dehydrogenase 1, *Sox2* Sex determining region Y (SRY)-box 2, *GAPDH* Glyceraldehyde 3-phosphate dehydrogenase, *F* Forward, *R* Reverse

### Statistical analyses

All statistical analyses were performed using statistical package for the social sciences version 19.0. (IBM Inc., USA). Continuous variables are presented as means ± standard deviations. The differences between multiple groups were analyzed using a one-way analysis of variance, followed by a post-hoc least significance difference test. An independent *t*-test was used to compare differences between groups, and two-sided *P*-values < 0.05 were considered statistically significant.

## Results

### SP cells formed subpopulation in MM cell lines

**Fig. 1:**
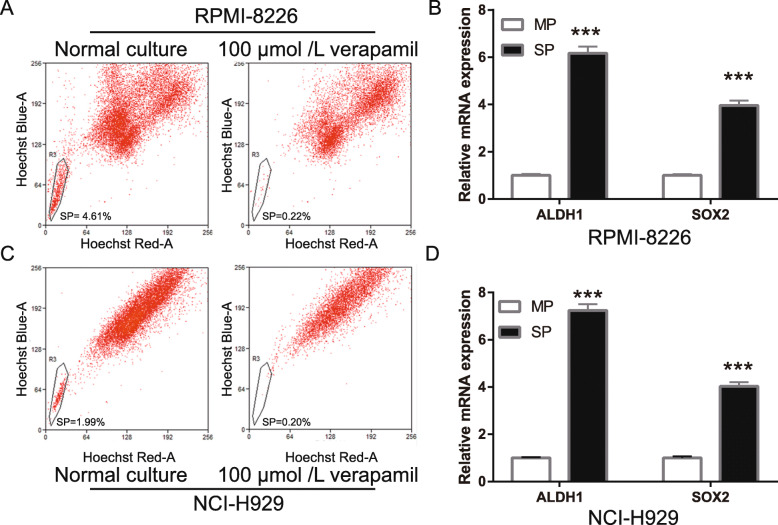
Side population (SP) cells form subpopulation in multiple myeloma (MM) cell lines. **a** and **c** SP cells were isolated in RPMI-8226 and NCI-H929 cells using Hoechst 33342 fluorescence staining method with fluorescence-activated cell sorting (FACS). **b** and **d** Expression of SP cell surface markers including aldehyde dehydrogenase 1 (ALDH1) and sex determining region Y (SRY)-box 2 Sox2 were measured using quantitative reverse transcription-polymerase chain reaction (qRT-PCR). ***P < 0.001

To investigate the effect of cotreatment with DATS+Dex on SP cells in MM, SP cells were isolated from RPMI-8226 and NCI-H929 cells using the Hoechst 33342 fluorescence-activated cell sorting. The results showed that the number of SP cells in RPMI-8226 and NCI-H929 cells was 4.65 ± 0.125 and 1.99 ± 0.138, respectively (Fig. 1a and c). Then, the expression of the SP cell surface markers, aldehyde dehydrogenase 1 (ALDH1) and sex determining region Y (SRY)-box 2 (Sox2), was measured using qRT-PCR. The mRNA expression of ALDH1 and Sox2 in SP cells was significantly higher than that in MP cells (Fig. 1b and d). These results showed that SP cells were successfully separated from MM cells.

### Cotreatment with DATS+Dex inhibited proliferation of MM SP cells and colony formation

**Fig. 2:**
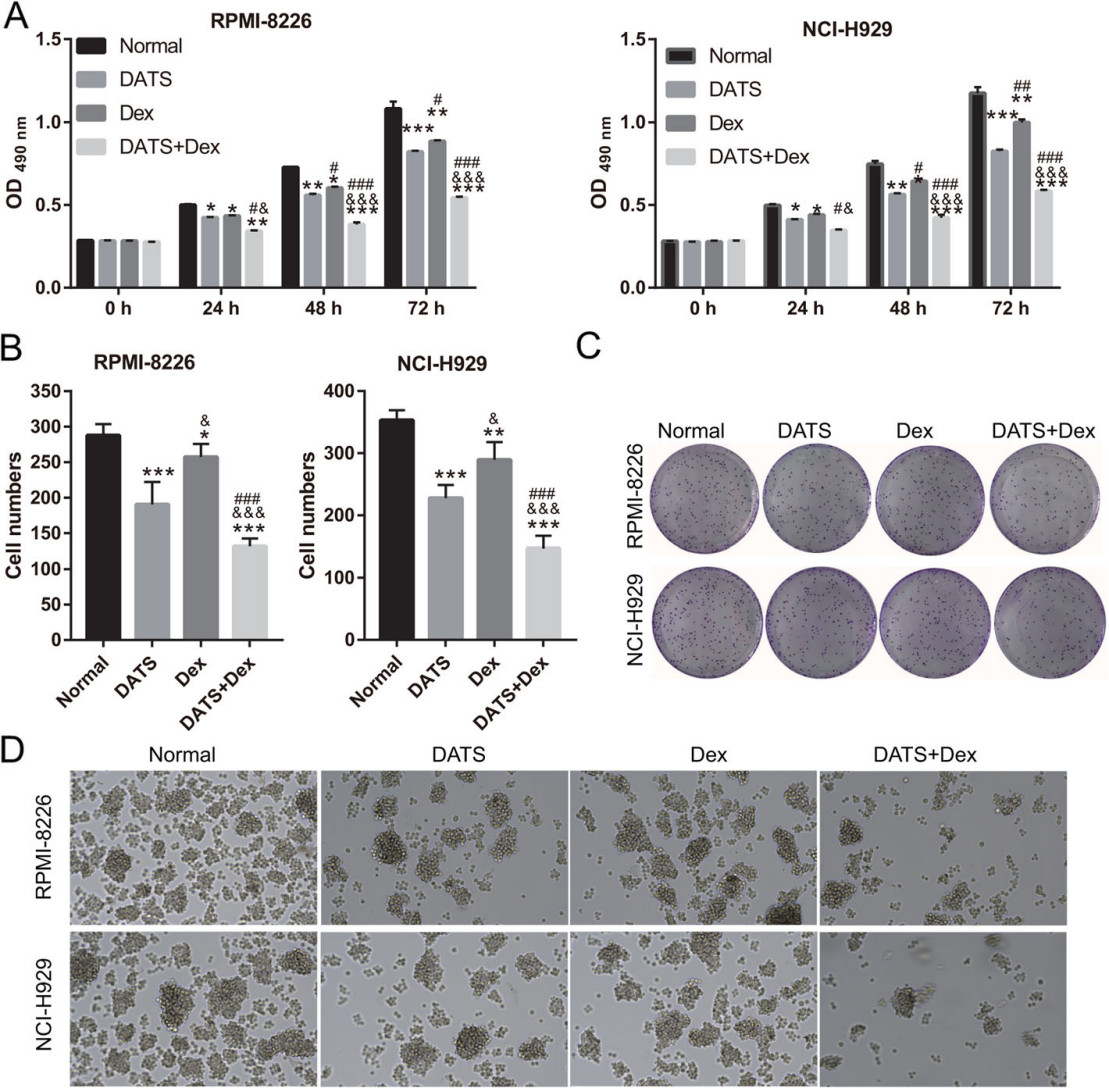
Diallyl thiosulfinate and dexamethasone (DATS+Dex) cotreatment inhibited proliferation and colony formation of multiple myeloma (MM) side population (SP) cells. **a** Effect of DATS, Dex, and DATS+Dex treatments on proliferation of MM SP cells detected using MTS analysis at 0, 24, 48, 72 h after treatment. **b** Bar represents cell numbers of colonies formed in SP cells after treatment with DATS, Dex, or DATS+Dex. **c** Representative image of colony formation by SP cells after treatment with DATS, Dex, or DATS+Dex. **d** Representative image of spheroids formation by SP cells after treatment with DATS, Dex, or DATS+Dex (magnification, × 100). ***P < 0.001 vs normal group, ^&^*P* < 0.05 and ^&&&^*P* < 0.001 vs DATS group, and ^###^*P* < 0.001 vs Dex

To investigate whether DATS+Dex affected the survival rate of MM SP cells, cells proliferation was analyzed using MTS assay as well as for colony formation and spheroid formation after drug treatment (Fig. 2). We observed that the proliferation, colony formation, and spheroid formation of SP cells were significantly lower in cells treated with DATS, Dex, or DATS+Dex than in untreated cells. Proliferation, colony formation, and spheroid formation in the DATS-treated group were lower than those in the Dex-treated group. Additionally, proliferation, colony formation, and spheroid formation following DATS+Dex cotreatment were significantly lower than those following DATS or Dex treatment alone. These results suggested that cotreatment with DATS+Dex inhibited the proliferation and spheroid formation of MM SP cells more effectively than either agent did alone.

### Cotreatment with DATS+Dex inhibited cell cycle and promoted apoptosis of SP cells

**Fig. 3:**
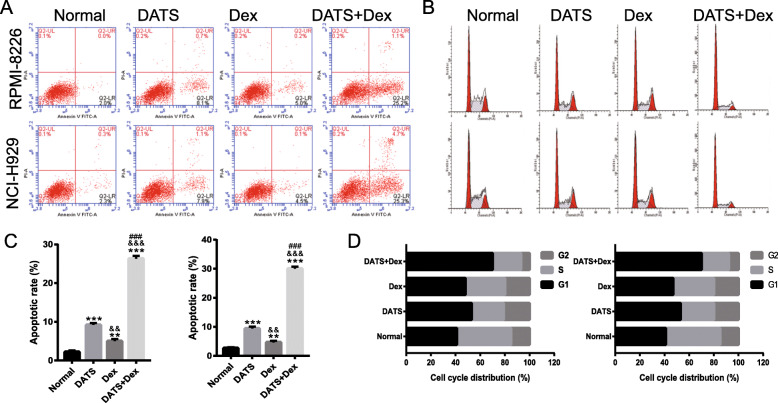
Diallyl thiosulfinate and dexamethasone (DATS+Dex) cotreatment treatment promoted apoptosis of multiple myeloma (MM) side population (SP) cells and arrested their cell cycle. **a** and **b** Representative image of apoptosis and cell cycle of MM SP cells detected using flow cytometry analysis 48 h after treatment. Bar represents **c** apoptosis and **d** cell cycle distribution of MM SP cells. **P < 0.01 and ***P < 0.001 vs normal group, ^&&^*P* < 0.01 and ^&&&^*P* < 0.001 vs DATS group, and ^###^*P* < 0.001 vs Dex group

To elucidate the effect of cotreatment with DATS+Dex on SP cells, apoptosis and the cell cycle were evaluated after treatment with DATS, Dex, or DATS+Dex. Flow cytometry analysis revealed that the apoptosis rates following treatment with DATS, Dex, or DATS+Dex were significantly higher than those following no treatment. Apoptosis rates in the DATS group were higher than those in the Dex group, but apoptosis rate following cotreatment with DATS+Dex was much higher than that following treatment with DATS or Dex (Fig. 3a and c). In addition, the proportion of cells undergoing cell cycle arrest in the G1/S phase was altered. Compared with the untreated control group, DATS, Dex, or DATS+Dex groups showed a significantly higher percentage of cells in the G1-phase, which prevented their transition from G1 to S phase. The effect of DATS treatment was not significantly different from that of Dex treatment. Furthermore, DATS+Dex cotreatment markedly increased percentage of cells in the G1-phase than DATS or Dex treatment alone (Fig. 3b and d). These results suggest that cotreatment with DATS+Dex was more effective on apoptosis and cell cycle arrest in MM SP cells than either agent alone.

### Cotreatment with DATS+Dex inhibited PI3K/AKT/mTOR signaling in SP cells

**Fig. 4:**
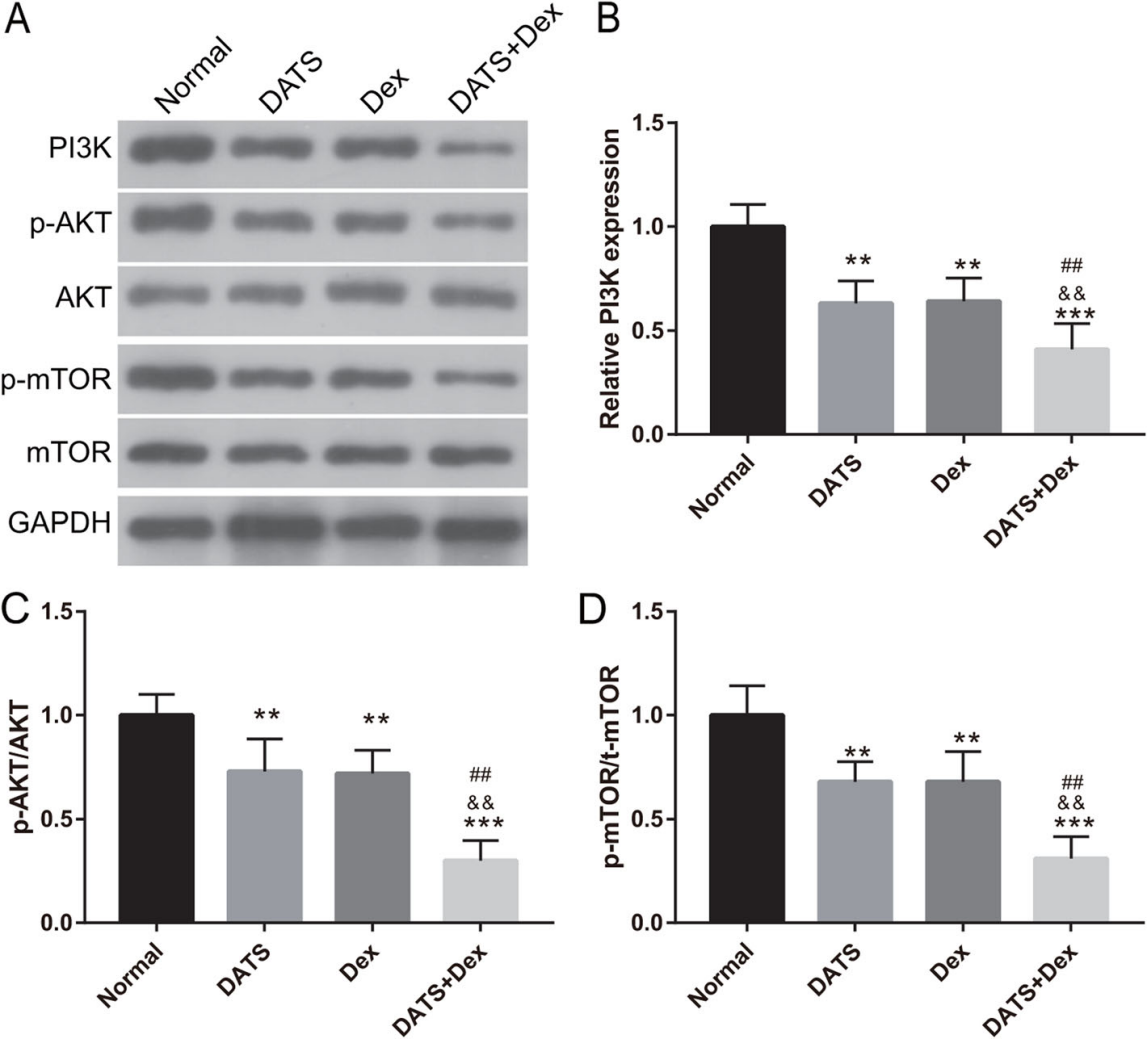
Diallyl thiosulfinate and dexamethasone (DATS+Dex) cotreatment silenced phosphoinositide 3 kinase (PI3K)/AKT/mechanistic target of rapamycin (mTOR) signaling in multiple myeloma (MM) side population (SP) cells. **a** Expression of PI3K, AKT, mTOR, phosphorylated mTOR (p-mTOR), and p-AKT was evaluated using western blotting. **b**–**d** Bar represents the expression of PI3K, p-AKT/AKT, p-mTOR/ mTOR. Note: Full-length blots gels are presented in Supplementary Figure 1. **P < 0.01 and ***P < 0.001 vs normal group, ^&&^*P* < 0.01 vs DATS group, and ^##^*P* < 0.01 vs Dex group

We further sought to explore whether cotreatment with DATS+Dex suppressed the PI3K/AKT/mTOR signaling pathway. The expression of the components of the PI3K/AKT/mTOR pathway in SP cells was detected using western blotting after treatment with DATS, Dex, or DATS+Dex (Fig. 4). The analysis showed that the expression of PI3K, p-AKT/AKT, and p-mTOR/mTOR was lower in MM SP cells treated with DATS, Dex, or DATS+Dex than that in untreated cells. Cotreatment with DATS+Dex clearly suppressed the protein levels of PI3K, p-AKT/AKT, and p-mTOR/mTOR more than treatment with DATS or Dex alone. Additionally, no significant differences in protein expression were observed between DATS and Dex treatments alone. These results indicated that cotreatment with DATS+Dex significantly inhibited PI3K/AKT/mTOR signaling in MM SP cells.

### Cotreatment with DATS+Dex promoted miR-127-3p expression in SP cells

**Fig. 5:**
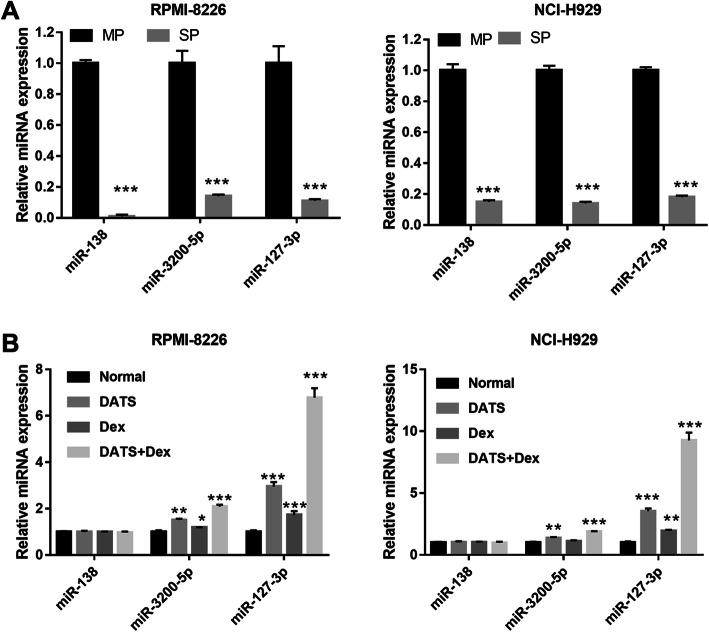
Cotreatment with diallyl thiosulfinate and dexamethasone (DATS+Dex) promoted miR-3200-5p and miR-127-3p expression. **a** miR-138, miR-3200-5p, and miR-127-3p expression was significantly lower in side population (SP) cells than in MP cells. Expression of all miRNAs in SP and MP cells of RPMI-8226 and NCI-H929 cell lines was measured using quantitative reverse transcription-polymerase chain reaction (qRT-PCR). **b** DATS, Dex, and DATS+Dex treatment significantly increased miR-3200-5p and miR-127-3p expression in MM SP cells, but did not affect miR-138 expression, compared with no treatment. *P < 0.05, **P < 0.01, and ***P < 0.001 vs normal group

To explore the mechanism underlying the inhibitory effects of DATS+Dex cotreatment on the proliferation of SP cells, we further analyzed the expression of miRNAs after treatment. The results from GSE56163 showed that hsa-miR-138, hsa-miR-3200-5p, and hsa-miR-127-3p levels were significantly higher in SP cells than in MP cells in MM. We then measured miR-138, miR-3200-5p, and miR-127-3p expression in the SP and MP cells of RPMI-8226 and NCI-H929 cells. The results showed that miR-138, miR-3200-5p, and miR-127-3p expression was significantly lower in SP cells than in MP cells (Fig. 5a). Additionally, we found that DATS, Dex, and DATS+Dex treatments induced significantly higher miR-3200-5p and miR-127-3p expression, especially of the latter, than control treatment in MM SP cells (Fig. 5). Therefore, we chose miR-127-3p for the further experiments.

### Silencing of miR-127-3p expression weakened DATS+Dex cotreatment effect

**Fig. 6:**
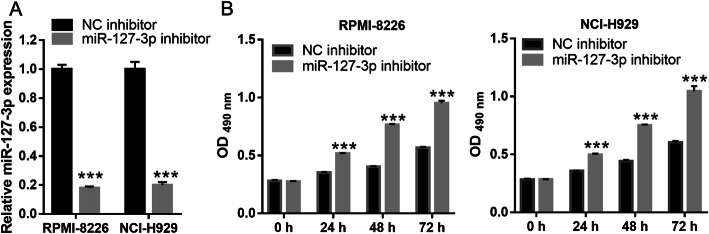
Silencing miR-127-3p expression reversed effect of cotreatment with diallyl thiosulfinate and dexamethasone (DATS+Dex) on proliferation of multiple myeloma (MM) side population (SP) cells. **a** miR-127-3p expression was inhibited in cells transfected with miR-127-5p. **b** Proliferation of MM SP cells detected using MTS analysis at 0, 24, 48, 72 h after treatment. Silencing miR-127-3p expression promoted proliferation of MM SP cells cotreated with DATS+Dex. ***P < 0.001

**Fig. 7:**
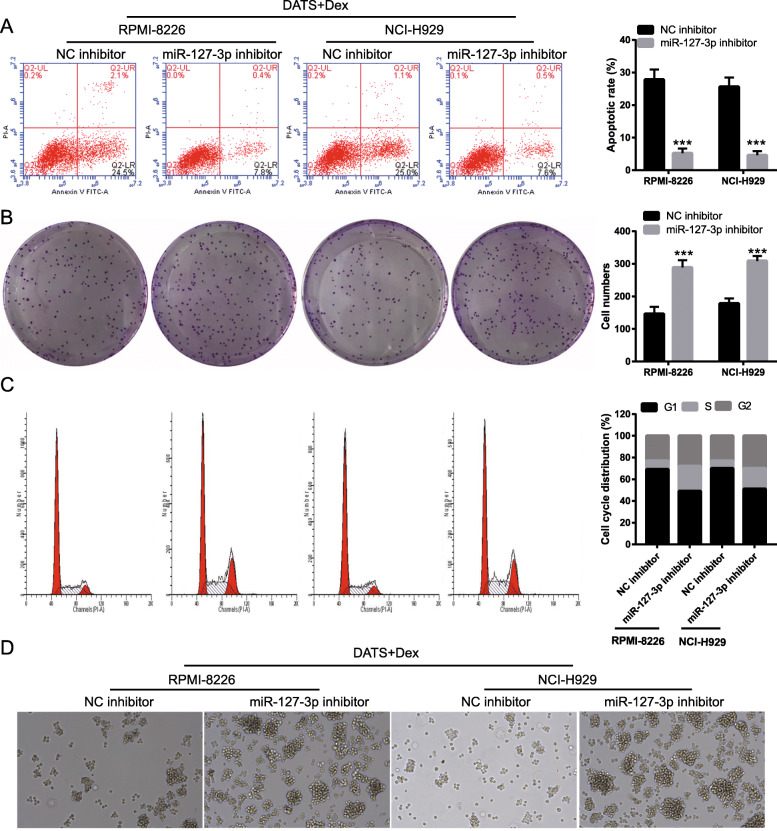
Silencing miR-127-3p expression reversed effect of cotreatment with diallyl thiosulfinate and dexamethasone (DATS+Dex) on the apoptosis, colony formation, cell cycle, and spheroids formation of multiple myeloma (MM) side population (SP) cells. Silencing miR-127-3p expression **a** inhibited apoptosis, **b** promoted colony formation, and **c** promoted cell cycle progression of MM SP cells treated with DATS+Dex. **d** Representative image of spheroids formation by SP cells after treatment with DATS, Dex, or DATS+Dex (magnification, × 100). ***P < 0.001

**Fig. 8:**
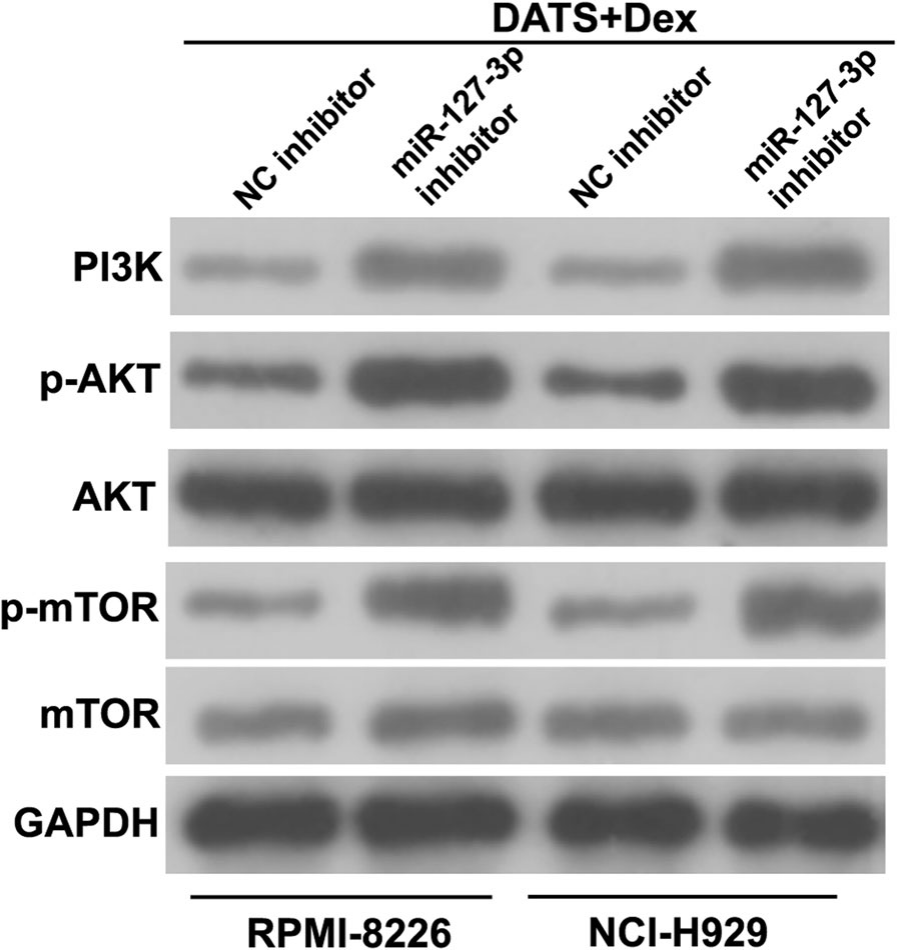
Silencing miR-127-3p expression reversed effect of cotreatment with diallyl thiosulfinate and dexamethasone (DATS+Dex) on phosphoinositide 3-kinase (PI3K) pathway of multiple myeloma (MM) side population (SP) cells. Expression of PI3K, phosphorylated AKT (p-AKT)/AKT, and p-mechanistic target of rapamycin (mTOR)/mTOR in MM SP cells analyzed using western blotting. Note: Full-length blots gels are presented in Supplementary Figure 2

Next, we explored whether cotreatment with DATS+Dex inhibited the proliferation of SP cells by regulating miR-127-3p. First, qRT-PCR of MM SP cells transfected with an miR-127-3p inhibitor showed that miR-127-3p expression was inhibited after transfection (Fig. 6a). Additionally, silencing miR-127-3p significantly promoted the proliferation of MM SP cells treated with DATS+Dex (Fig. 6b). The flow cytometry assay indicated that silencing miR-127-3p significantly inhibited cell apoptosis and promoted cell cycle progression and colony formation in MM SP cells treated with DATS+Dex (Fig. 7). As shown in Fig. 8, silencing miR-127-3p increased the expression of PI3K, p-AKT/AKT, and p-mTOR/mTOR in MM SP cells treated with DATS+Dex. These results suggest that silencing miR-127-3p weakened the effect of DATS+Dex cotreatment on the proliferation of SP cells.

## Discussion

DATS is a herbal drug that has long been known for its medicinal properties [19], including antitumor activity [7, 8]. Gao et al. [20] reported that DATS enhances the efficiency of cyclophosphamide in the treatment of neuroblastoma. Wang et al. [21] have reported that recombinant interleukin-2 plus DATS cotreatment suppresses pancreatic cancer. In our previous study, we found that DATS inhibited the proliferation and colony formation of MM SP cells, inducing G1/S arrest [9]. In this study, we found that compared with DATS or Dex treatment alone, cotreatment with DATS+Dex resulted in greater inhibition of colony formation, spheroids formation, and proliferation of SP cells and in greater induction of apoptosis and cell cycle arrest in the G1/S phase. These results suggest that cotreatment with DATS+Dex inhibited proliferation and promoted apoptosis of MM SP cells more effectively than treatment with DATS or Dex alone.

miRNAs are emerging as important modulators of cellular signaling, including cell proliferation, in MM. In this study, we found that miR-127-3p expression was more significantly inhibited in MM SP cells than it was in MM MP cells. A previous study found that miR-127-3p expression was significantly reduced and that the miRNA acted as tumor suppressors in gastric, prostatic, and ovarian cancers [22–24]. In this study, we found that treatment with DATS and Dex alone or in combination, significantly promoted miR-127-3p expression and that silencing miR-127-3p reversed the effect of these treatments on the proliferation of SP cells. These results suggest that miR-127-3p acts as a tumor suppressor in MM, similar to that in other cancers. DATS+Dex cotreatment inhibited the proliferation of MM SP cells by upregulating miR-127-3p expression.

The PI3K/AKT/mTOR signaling pathway is known to control cell survival and is abnormally activated in a wide variety of cancers, resulting in inhibition of apoptosis via multiple mechanisms [25]. Recent studies have shown that the activation of the PI3K/AKT/mTOR signaling pathway plays a key role in the survival and proliferation of MM cells [26, 27]. In the present study, a combination of DATS+Dex decreased the expression of PI3K, p-AKT/AKT, and p-mTOR/mTOR, suggesting that DATS+Dex cotreatment deactivated the PI3K/AKT signaling pathway. Additionally, silencing miR-127-3p activated the PI3K/AKT signaling pathway and reversed the effect of DATS and Dex treatment alone or in combination on the PI3K/AKT signaling pathway. These results showed that cotreatment with DATS+Dex negatively regulated the PI3K/AKT/mTOR signaling pathway to exert anticancer effects in MM SP cells through upregulating miR-127-3p expression.

## Conclusion

Cotreatment with DATS+Dex inhibited the proliferation of MM SP cells by promoting miR-127-3p expression and deactivating the PI3K/AKT signaling pathway. Our data provide a theoretical basis for the clinical application of DATS and Dex in MM patients. However, further research is needed to investigate these effects in animal models and clinical trials.

## Supplementary Information

**Additional file 1.**

**Additional file 2.**

## Data Availability

The datasets used, analyzed, or both during the current study are available from the corresponding author upon reasonable request.

## Abbreviations

MM: Multiple myeloma
SP: Side population
MP: Main population
Dex: Dexamethasone
DATS: Diallyl thiosulfinate
miRNAs: MicroRNAs
