# Supplementary figures and images for: Diallyl thiosulfinate enhanced the anti-cancer activity of dexamethasone in the side population cells of multiple myeloma by promoting miR-127-3p and deactivating the PI3K/AKT signaling pathway
Source: BMC Cancer. 2021 Feb 6;21:125. doi: 10.1186/s12885-021-07833-5 (PMC7866463; doi:10.1186/s12885-021-07833-5)

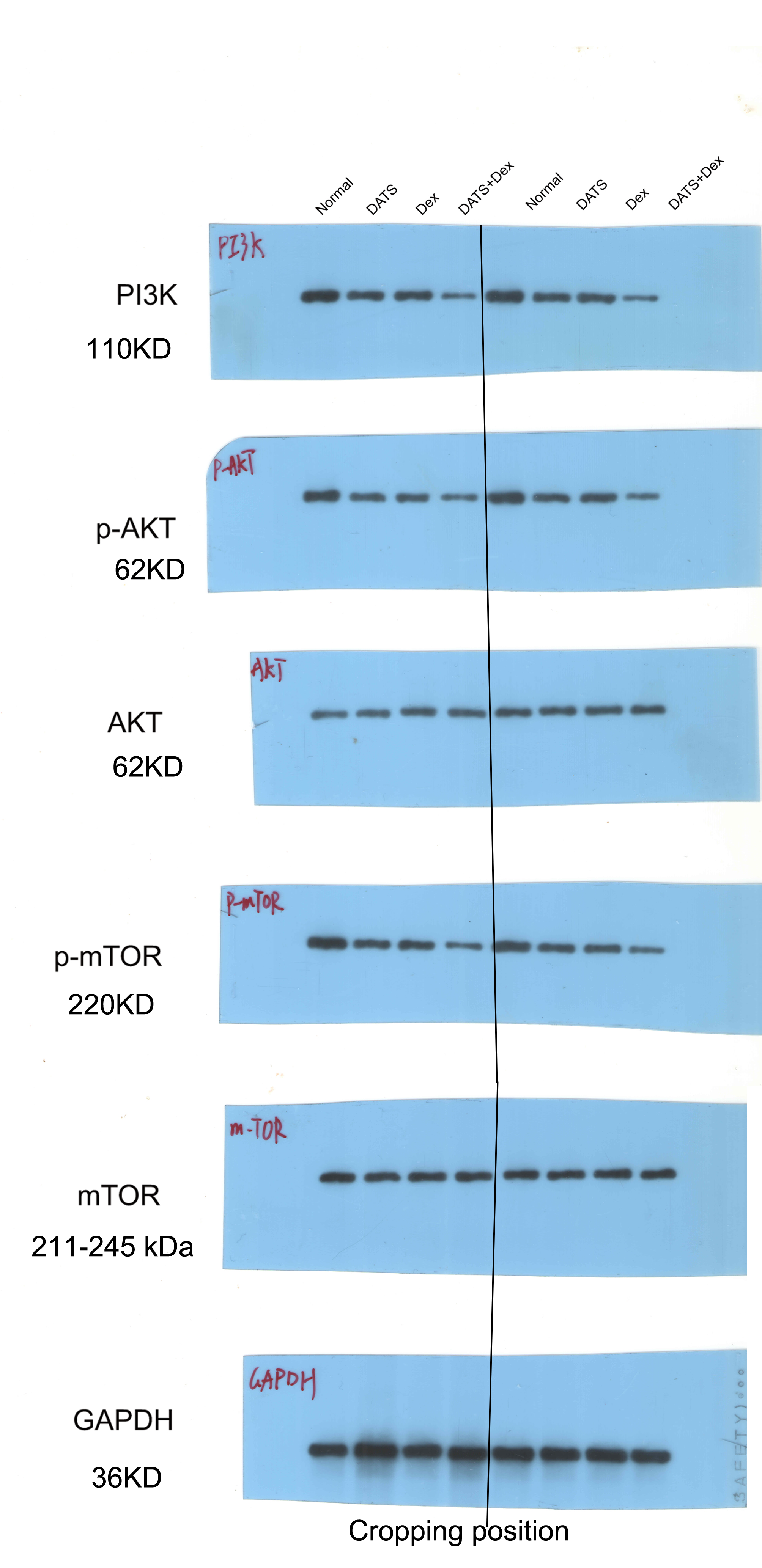

Supplement: Supplementary file 1 — Additional file 1. [file 12885_2021_7833_MOESM1_ESM.tif]

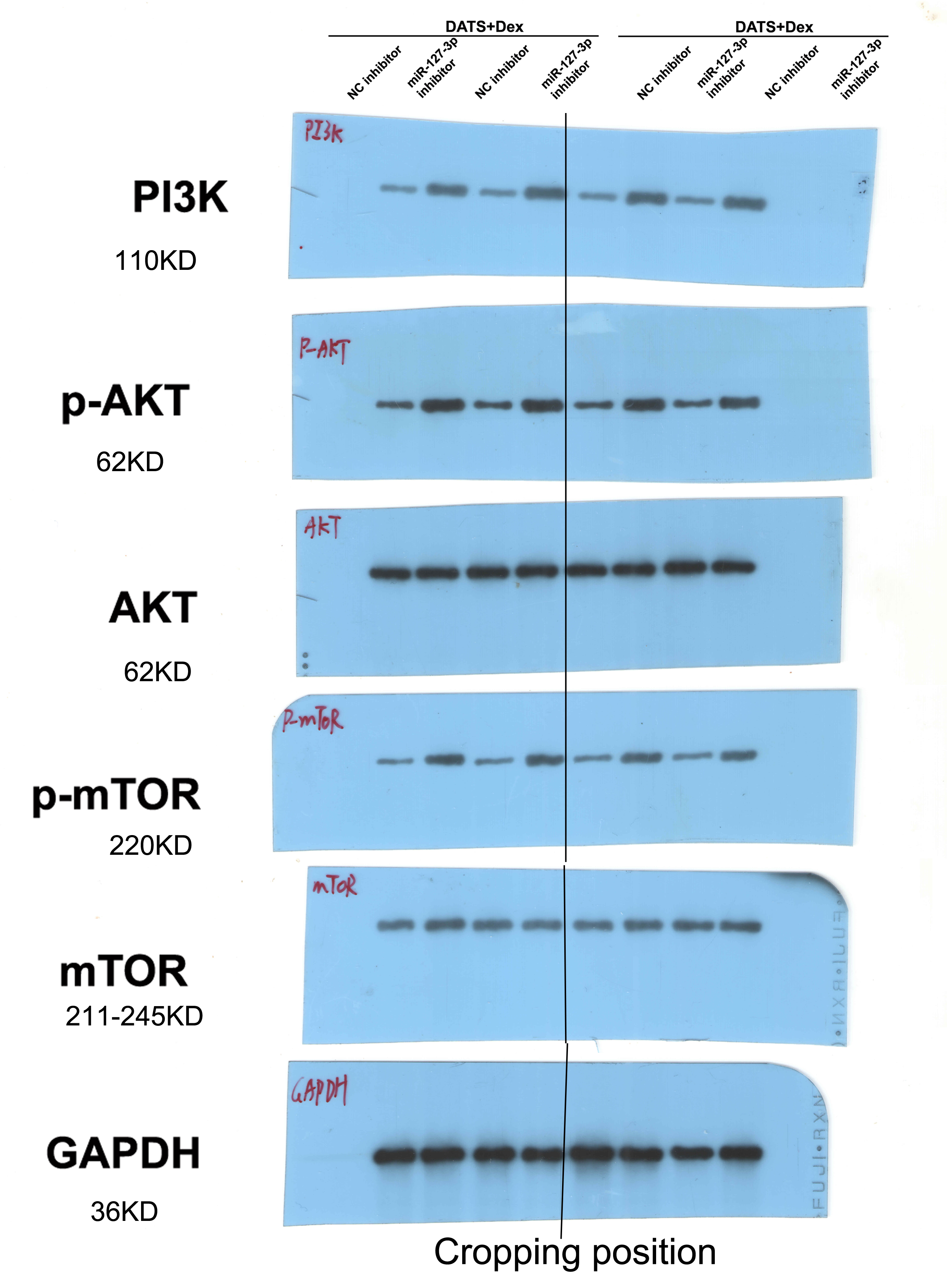

Supplement: Supplementary file 2 — Additional file 2. [file 12885_2021_7833_MOESM2_ESM.tif]
